# Impact of Percutaneous Mitral Valve Repair on Left Atrial Strain and Atrial Fibrillation Progression

**DOI:** 10.3390/jcdd10080320

**Published:** 2023-07-28

**Authors:** Letizia Rosa Romano, Giuseppe Scalzi, Biagio Malizia, Iolanda Aquila, Alberto Polimeni, Ciro Indolfi, Antonio Curcio

**Affiliations:** 1 Division of Cardiology, Department of Medical and Surgical Sciences, Magna Graecia University, 88100 Catanzaro, Italy; 2 Division of Cardiology, Department of Pharmacy, Health and Nutritional Sciences, University of Calabria, 87100 Cosenza, Italy

**Keywords:** atrial fibrillation, speckle-tracking echocardiography, atrial cardiomyopathy, TEER, cath lab

## Abstract

Transcatheter edge-to-edge repair (TEER) currently represents a valuable therapeutic option for patients with severe mitral regurgitation (MR) considered at high surgical risk. Besides symptoms and left ventricular (LV) echocardiographic improvements upon TEER, it has been postulated that left atrial (LA) function plays a prognostic role. The aims of our study were to evaluate LA changes after TEER, measured by two-dimensional speckle-tracking echocardiography analysis (2D-STE), their association with atrial fibrillation (AF) occurrence, and relative arrhythmic burden. We considered in a single-center study 109 patients affected by symptomatic severe MR undergoing TEER from February 2015 to April 2022. By 2D-STE, LA reservoir (R_s), conduct (D_s), and contractile (C_s) strains were assessed along with four-chamber emptying fraction (LAEF-4CH) before, 1, 6, and 12 months following TEER. Statistical analysis for comparison among baseline, and follow-ups after TEER was carried out by ANOVA, MANOVA, and linear regression. Successful TEER significantly improved LV dimensions and LA performances, as indicated by all strain components, and LAEF-4CH after 1 year. Strikingly, a significant reduction in arrhythmic burden was observed, since only one case of subclinical AF detected by a previously implanted cardiac electronic device was found in the cohort of sinus rhythm patients (*n* = 48) undergone TEER; in addition, ventricular rate was reduced in the AF cohort (*n* = 61) compared to baseline, together with few episodes of nonsustained ventricular tachycardias (5/61, 8.2%) after MR improvement. Overall, TEER was associated with improved cardiac performance, LA function amelioration, and reduced arrhythmic burden.

## 1. Introduction

Mitral regurgitation (MR) is an abnormal systolic reversal of blood from the left ventricle (LV) into the left atrium (LA) due to incomplete closure of the mitral valve [1]. It represents the second most common valve disease in Europe after aortic stenosis, and its incidence is increasing due to the aging of the population [2]. Percutaneous mitral valve repair through transcatheter edge-to-edge repair (TEER) is widely demonstrated to be able to reduce the grade of MR in patients at high surgical risk [3,4]. In the MR untreated scenario, the development of LA cardiomyopathy has been demonstrated to occur, as it progressively determines cellular and structural alterations that predispose to the development of atrial fibrillation (AF) [5,6,7]. Similarly, LV assumes a more spherical shape, with broad LV wall stress and end-diastolic pressure, hence leading to a decreased contractile state, with reduced myofiber content and interstitial fibrosis [8,9,10]. As this process continues, irreversible LV dysfunction occurs, which leads to the decompensated stage of MR with symptoms of heart failure (HF) and ventricular arrhythmias as well [11].

However, early recognition, classification of etiology, and appropriate timing of critical interventions in patients presenting with suspected severe MR may result in better long-term outcomes [12,13,14]. The beneficial effects of improved LV geometry and LA shape have not been extensively investigated by far; in this regard, the restoration of sinus rhythm in patients with AF has shown an improvement in MR severity [15], suggesting a causal relationship between MR and AF, but the selective impact on LA function and the following arrhythmic burden are yet matters of discussion.

Further than staging MR, echocardiography might provide important advancements in understanding LA deterioration and the development of atrial cardiomyopathy [9,16]. More in detail, speckle-tracking echocardiography (STE) offers a quantitative assessment that is less load-dependent compared to traditional parameters of LA function [17].

LA remodeling is a response of cardiomyocytes to various stressors including MR that causes volume overload. In this circumstance, both structural (increased interstitial fibrosis) and cellular (reduced action potential duration) changes occur. These alterations, together, promote nonuniform impulse propagation that is a substrate for AF maintenance [8,15].

Therefore, we conducted a study on the role of transcatheter mitral valve implantation in patients with severe MR and contraindications for surgery or high operative risk for addressing the beneficial effects on LA and LV geometries by reducing the atrial and ventricular arrhythmias occurrence.

## 2. Materials and Methods

### 2.1. Study Population

Moderate to severe and severe MR patients (*n* = 109) not amenable to surgical repair were enrolled at the Department of Cardiology of the Magna Graecia University of Catanzaro from February 2015 to April 2022 for the TEER procedure. The main inclusion criteria, according to the COAPT trial [14], were symptomatic HF in New York Heart Association (NYHA) functional class II, III or ambulatory IV; LV ejection fraction (LVEF) > 20%; LV diameter ≤ 70 mm; anatomy judged suitable for TEER. All patients provided written informed consent to the procedure after an interdisciplinary team of cardiologists, anesthesiologists, and cardiac surgeons confirmed TEER appropriateness based on EuroSCORE II and on other surgical risk factors not included in the score itself [18]. Ongoing guidelines-directed medical therapy was mandatory, as well as percutaneous angioplasty/stenting and implantable cardioverter defibrillator/cardiac resynchronization therapy devices prior to TEER, when needed. Baseline functional status and follow-up were assessed according to the NYHA classification. The study was conducted in accordance with the Declaration of Helsinki and approved by the institutional review board.

### 2.2. Echocardiographic Assessments

All patients underwent standard transthoracic echocardiography using Vivid 9, Vivid E 95, or Vivid S70 ultrasound systems (GE Vingmed, Milwaukee, WI, USA) equipped with a 3.5 mHz M5S transducer. M-mode, 2D, color Doppler, pulsed-wave, and continuous-wave Doppler data were stored on a dedicated workstation for off-line analysis with “EchoPAC GE Healthcare Software Only, Version 204.73”.

Calculations were performed according to American Society of Echocardiography guidelines [19,20]. All patients were assigned an MR severity score of 1 (mild), 2 (mild to moderate), 3 (moderate to severe), or 4 (severe) according to the quantitative measurements of the effective regurgitant orifice area (EROA) and regurgitant volume (RgV). RgV was estimated as the EROA multiplied by the integral time velocity of the regurgitant jet (VTI). LVEF was obtained from two- and four-chamber apical views using the modified Simpson rule. End-diastolic and end-systolic diameters (EDD and ESD, respectively) were measured at the LV minor axis, approximately at the tips of the mitral valve leaflets, in the parasternal view, using bidimensional echocardiography. Speckle-tracking analyses were performed with EchoPAC software. Apical four-chamber views were acquired using standard 2D grayscale echocardiography during breath hold and with a stable ECG recording. To ensure optimal quality, three consecutive cardiac cycles were acquired at a rate of 50–80 frames/s in sinus rhythm or with ≤10% heart rate variability when patients were in AF.

The endocardial border of the LA was plotted excluding the appendage and pulmonary veins from the cavity, and the longitudinal elongation of the LA wall during systole was used for the analysis (LA systolic strain). More in detail, three components were further evaluated, namely LA reservoir strain (R_s), conduct strain (D_s), and contractile strain (C_s), together with the four-chamber emptying fraction (LAEF-4CH); the “reservoir” aspect corresponds to the LV systole; the “conduit” function Is identified when blood runs from LA to LV during early diastole; and the “contractile” feature represents the atrial systole. In patients with permanent AF, the lack of productive atrial systole did not allow D_s and C_s assessments [17]. Finally, LAEF-4CH was calculated with the following formula: (LA maximum volume–LA minimum volume)/LA maximum volume × 100%. All parameters were acquired before, 1, 6, and 12 months following TEER.

Exclusion criteria were evidence of structural mitral valve disease (contemporary presence of severe MR and rheumatic mitral valve disease, extensive mitral valve prolapse and a greater extent leaflet calcification or extensive annular calcification), association with other valvular diseases, diagnosis of hypertrophic or idiopathic dilated cardiomyopathies, congenital heart diseases, pulmonary embolism, and constrictive pericarditis.

### 2.3. Transcatheter Edge-to-Edge Repair

TEER was performed with MitraClip™ (Abbott Vascular Structural Heart, Santa Clara, CA, USA) according to the standard procedure under general anesthesia [21]. The interventions were performed under fluoroscopic visualization and by transesophageal echocardiography (TEE) guidance. MR was handled in the majority of cases with one clip; the need for additional clips was addressed according to specific technical and clinical circumstances that were shared by physicians involved in the cath lab. Briefly, after a demonstration of the degree of MR reduction and the severity of residual MR, discussion and agreement as to the internal protocol were reached based on the transmitral valve mean gradient, valve area, improvement in hemodynamics, and technical feasibility of placing a second clip; rarely, three clips were required.

Procedural success was defined from the implantation of at least one clip and reduction of severity of MR ≤ 2+ assessed at intraprocedural TEE.

**Figure 1:**
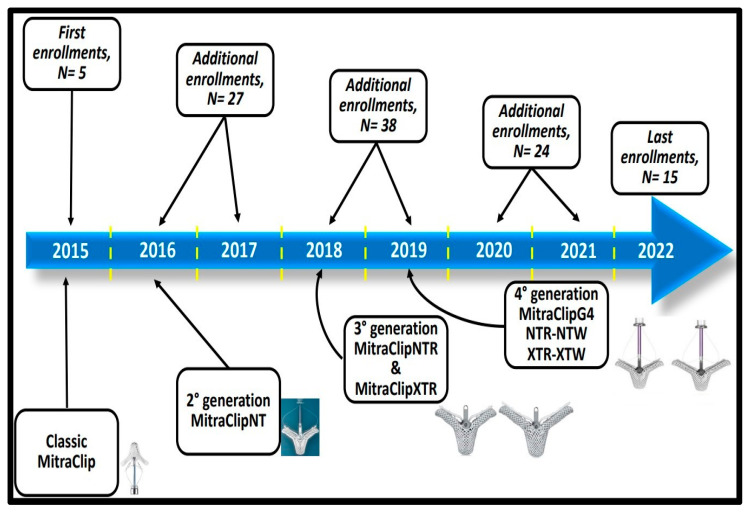
Timeline of the enrolled population. The reported timeline indicates the total cohort of patients enrolled, according to device availability (generations depicted, bottom rectangles), and timings of recruitment (upper rectangles).

The choice of devices to be deployed (Figure 1) was made according to the availability of production. Standard therapy was maintained after discharge, and dual antiplatelet therapy following TEER was added according to current recommendations [22], as well as oral anticoagulants when needed.

### 2.4. Cardiac Rhythm Assessments

Standard electrocardiography (ECG) and 24 h ambulatory ECG monitoring were performed in all cases accomplishing state-of-the-art requirements [23]. When available, coronary care unit telemetric monitoring was checked for heart rate (HR) and cardiac arrhythmias occurrence. Basically, perturbations of the basal sinus rhythm (*n* = 48 patients, Table 1) were investigated, including AF occurrence and/or ventricular arrhythmias considered as ventricular extrasystolic beats, nonsustained ventricular tachycardias (NSVT), and VT. Moreover, patients in sinus rhythm previously implanted with pacemakers or implantable cardioverter/defibrillator, or cardiac resynchronization therapy devices with a defibrillator (Table 1) having an atrial lead were addressed during outpatient visits or by means of remote device monitoring for atrial high-rate episodes (AHREs) findings. AF was classified as paroxysmal, persistent, or permanent, as reported in current guidelines [24]. Data on heart rate ventricular response for persistent and permanent forms were also extracted. “AF burden” was defined as the overall time spent in AHRE/subclinical AF during the prespecified period of observation of 24 h. In order to ensure the homogeneity of results, we considered an equal monitoring period in all AF patients.

### 2.5. Statistical Analysis

Continuous variables are presented as mean ± standard deviations (SD) and categorical variables as values and percentages. The Shapiro–Wilk test was used to assess the normality of continuous variables. Comparisons between atrial strain’s components were performed with an analysis of variance (ANOVA) test. Multivariate analysis of variance (MANOVA) was assessed to determine if AF was impacted by atrial function. Bonferroni’s test for multiple comparisons was conducted as a post hoc analysis. The correlation between AF burden and LA function was addressed using Pearson’s analysis. Linear regression analysis predicted the relationship between atrial strains and arrhythmic burden. A bilateral value of *p* < 0.05 was considered significant. All data were processed using the Statistical Package for Science Social, Version 23 (SPSS, Chicago, IL, USA).

## 3. Results

### 3.1. Baseline Characteristics

One hundred and thirty-eight patients who had undergone TEER were considered for enrollment. Of these, 29 patients were excluded for the poor quality of echo acquisition images; hence, 109 patients were included in the final cohort. Patient recruitment, according to device availability, is shown in Figure 1. The learning curve was not modified depending on device updates and new market releases (three generations of TEER devices have been introduced in the clinical setting in recent years), nor was it affected by COVID-19 restrictions in patient admissions and cath lab access as it was during the pandemic. Clinical indication for TEER was derived from a high EuroSCORE II risk assessment (11.0 ± 11.3) for all patients who were admitted while under clinical control according to guideline-directed medical therapy and NYHA Class II–III. The proportion of patients with HF and reduced EF (hFrEF) was 43% (*n* = 47). The other baseline characteristics of the study population are shown in Table 1.

### 3.2. Procedural Outcomes

**Figure 2:**
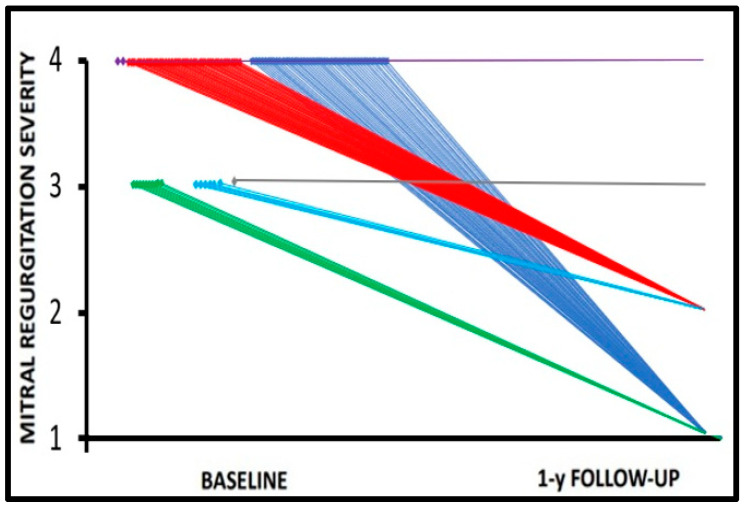
Stable mitral regurgitation amelioration following TEER. Linear projection over time showing MR improvement assessed at one year after transcatheter edge-to-edge repair. A dramatic reduction of MR from 4+ to 1+ (blue) or 2+ (red) grade and from 3+ to 1+ (green) or 2+ (light blue) grade was observed in 95.4% of enrolled patients; on the contrary, 2.7% of cases remained in MR 3+ (gray) and 1.8% in MR 4+ (violet). Long-term results paralleled the MR severity at discharge.

Despite the different etiologies of MR (Table 1), TEER was associated with significant amelioration in both RV and LV functions. A slight increase in LVEF (baseline: 44.1 ± 12.0%; 1-year follow-up: 45.9 ± 11.0%, *p* = 0.04) was accompanied by amelioration in global longitudinal strain (−13.7 ± 7.9% at baseline vs. GLS: −15.0 ± 7.9% at follow-up, *p* = 0.04), and RV GS was increased as well (baseline: −13.0 ± 6.0%; follow-up: −15.5 ± 4.1%, *p* = 0.04). All the reported improvements, together with the additional parameters reported in Table 2, are mainly dependent on the reduction in MR severity; remarkably, postoperative MR ≤ 2 was achieved in 95.4% (*n* = 104) of patients; 2.8% (*n* = 3) of patients had a residual MR of 3+, and 1.8% (*n* = 2) of patients had a residual MR of 4+. The grade of MR before TEER was 3+ in 14.7% (*n* = 16) of patients and 4+ in 85.3% (*n* = 93). There were no relevant intraprocedural complications. The overall distributions of MR severity from baseline to discharge and similar at 1-year follow-up are shown in Figure 2. Major adverse cardiovascular events were experienced in 9 patients at 12 months; severe bleeding and/or transfusion were required in 2.7% (*n* = 3) of patients at 12 months. One case of partial prosthesis detachment (0.9%) was observed, and reintervention was not necessary as this study was ongoing.

### 3.3. Changes in Left Atrial Function

The design of this study composed of multiple follow-up points demonstrated that LA geometry and function proceed through a dynamic phenomenon. In fact, there were significant differences between all the follow-up windows for R_s [F (3, 227) = 12.57; *p* < 0.001]. More in detail, in the post hoc analysis, R_s (baseline 10.7 ± 5.7%) showed a major significant improvement after 12 months’ follow-up (16.3 ± 6.9%; *p* < 0.001). Similar results were obtained for D_s [F (3, 218) = 5.08; *p* = 0.001], e.g., in the post hoc analysis, from −6.7 ± 4.4% at baseline to −9.7 ± 4.5% at 12 months follow-up; *p* = 0.008. On the other hand, C_s did not show significant variation between groups [F (3, 191) = 2.24; *p* = 0.085], but in the post hoc analysis, there was a slight improvement, although it did not reach a significant difference between baseline −5.2 ± 5.1% vs. 12 months’ follow-up −7.8 ± 5.3%; *p* = 0.11. There was also a clear improvement of LAEF−4CH [F (3, 169) = 7.31; *p* < 0.001]. In post hoc analysis, it was more evident between baseline and 12 months’ follow-up (29.9 ± 14.1% vs. 36.5 ± 18.1%; *p* < 0.001). Multivariate analysis provided differences in basal R_s based on patient rhythm, F (10, 345) = 3.11, *p* = 0.006; Wilk’s Λ = 0.114, partial η2 = 0.515. In post hoc tests, a significant difference was found between sinus rhythm vs. AF permanent subgroups at baseline (15.2 ± 2.6% vs. 6.9 ± 3.23; *p* = 0.005), at 1 month (12.8 ± 3.9% vs. 5.1 ± 2.5%; *p* = 0.009), at 6 months (21 ± 6.8% vs. 7.6 ± 2.2%; *p* = 0.002), and at 12 months (21.6 ± 2.7% vs. 10.1 ± 4.3%; *p* = 0.002) follow-ups.

### 3.4. Atrial Fibrillation Progression and Arrhythmic Burden

**Figure 3:**
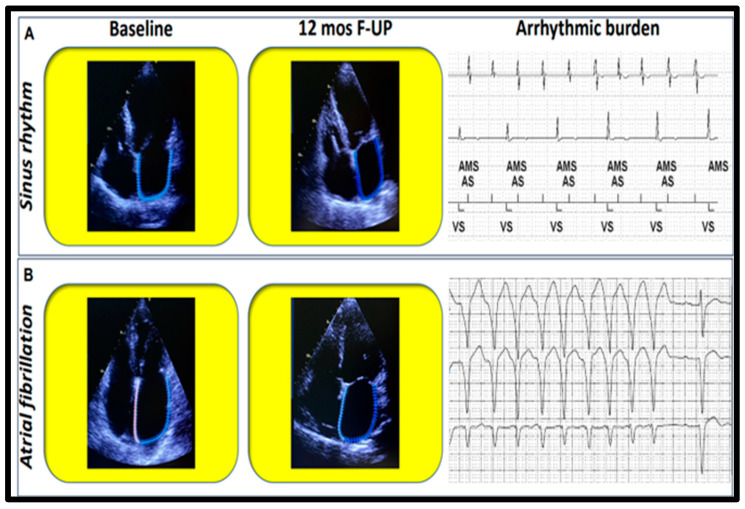
Arrhythmic burden after successful TEER: Correlation between LA strain reservoir (R_s) improvement and reduction of the arrhythmic burden. (**A**) AHRE, atrial high-rate episode, stored by previously implanted cardiac resynchronization therapy defibrillator, in a representative sinus rhythm patient; (**B**) NSVT, nonsustained ventricular tachycardia recorded at Holter ECG during follow-up visit at six months after transcatheter edge-to-edge repair in a typical patient from AF group.

To the permanent AF group (*n* = 34), no paroxysmal (*n* = 18) nor persistent case was added during follow-up after TEER. Average HR at follow-up was 69 ± 14 bpm for the entire enrolled population. In patients with paroxysmal AF, no progression to a persistent pattern was observed, while after intraprocedural cardioversion, sinus rhythm was reached and maintained in all patients (*n* = 9) that were affected by a persistent form. Mean CHA_2_DS_2_VASc was 4.5 ± 1.2. A total of 43 AHREs were detected at device interrogation of a patient in the sinus rhythm group (Figure 3), with a ventricular rate of 110 bpm, while AHRE average HR was 226 bpm. His CHA_2_DS_2_VASc score was 2, and accordingly, he underwent systematic screening for AF, without reaching AF diagnosis at this time.

Regarding arrhythmic burden, AF burden in paroxysmal AF (baseline: 17.6 ± 6.0 h, follow-up: 12.3 ± 6.4 h; *p* = 0.002) was significantly reduced as well as ventricular rate (baseline: 78 ± 12 bpm; 12 months’ follow-up: 73 ± 13 bpm; *p* = 0.006) in the permanent AF group (Table 3). This last result, as indicated by recent guidelines [25], refers to a good clinical response to pharmacologic and nonpharmacologic treatments, which in turn improves long-term outcomes.

There was a good correlation between paroxysmal AF burden and R_s: −0.808 (*p* < 0.0001). Linear regression demonstrated that R_s significantly predicted AF burden at baseline: the fitted regression model was AF burden = 25.6 − 0.8 * (R_s). The overall regression was statistically significant (R2 = 0.653, F (1, 17) = 31.9, *p* < 0.0001; β = −0.8, *p* < 0.0001).

NSVT episodes in AF patients were detected in 14.7% of cases (9/61) at baseline; their incidence was nearly halved (5/61, 8.2%) after MR improvement. Malignant ventricular arrhythmias such as sustained VTs or ventricular fibrillation were discovered in two patients from the AF group (3.3%) at baseline, without any repetition nor additional finding after MR transcatheter therapy.

## 4. Discussion

Since the first studies conducted, MR treatment with TEER has proven effective and safe, leading to a reduction in the severity of MR and an improvement in clinical status and quality of life in selected high-risk surgical patients [9,10].

Most likely, the reduction in the severity of the MR causes favorable hemodynamic changes in the left and right cardiac chambers, and such changes eventually affect clinical status and patient outcome [12,13,14].

The benefits resulting from TEER in terms of global improvement of cardiac function assessed by basal and advanced echocardiographic imaging modalities have been extensively described [26,27]. More in detail, it has been demonstrated that LA strain measurements offer the highest diagnostic accuracy, sensitivity, and specificity in predicting elevated LV filling pressures while providing a close figure of pulmonary capillary wedge pressure in HF [28]. Furthermore, the onset of paroxysmal AF can precede LA enlargement, hence reduced LA strain is the strongest independent echocardiographic predictor of progression from paroxysmal to persistent AF [6,7].

Since the selective contribution of the three components of the LA strain was not known in the progression of AF, we wanted to investigate whether some parameters could be considered more suggestive of having an advantage on cardiac rhythm stabilization. For these reasons, we addressed AF development during a long follow-up after TEER, whose suggestive goals were derived by intensive serial echocardiographic measurements in MR patients. The opportunity to address cardiac rhythm through telemetric device checks was further associated with cardiac rhythm monitoring through ambulatory Holter ECG. The net results were that AF developed in very few cases, as it was mostly asymptomatic (AHRE detection by previously implanted ICD, Figure 3), that patients affected by paroxysmal and persistent forms (*n* = 27) did not progress through permanent form, and that improved LA geometry was associated with somehow reduced AF average ventricular rate and reduced total burden as well (Table 3).

The LA strain has been shown to be useful in determining the effects of drugs on LA function in patients with systemic hypertension and HF [29]; nevertheless, the improvement of atrial transport function that can be obtained through the administration of angiotensin receptor (AR) blockers/angiotensin-converting enzyme inhibitors/AR-neprilysin inhibitors, beta-blockers, mineralocorticoid receptor antagonists, and sodium-glucose co-transporter-2 inhibitors in the HF setting cannot be translated entirely on atrial functional mitral regurgitation scenario [30]. On the other hand, a selective therapy on the LA such as ablation of AF, in spite of the different energy sources nowadays available, can increase LA fibrosis and, therefore, independently modify LA geometry and distensibility [31]. Numerous studies have evaluated the use of LA longitudinal strain in patients with asymptomatic MR of varying severity degrees and demonstrated that peak LA longitudinal strain is increased in patients with mild MR due to increased atrial compliance; in patients with moderate and severe MR, however, this index is decreased due to ultrastructural changes leading to interstitial fibrosis [11,17]. This demonstrates an inverse correlation between LA strain and fibrosis. The overload volume causes atrial remodeling (including increased fibrosis interstitial), a decrease in atrial elasticity, and a decline in LA reservoir function [28].

The main findings of the study indicate that TEER (1) improves LA function and (2) ameliorates longitudinal strain, and (3) LA strain assessment correlated with stabilization (in persistent and permanent AF) and even reduction (in paroxysmal AF) of the arrhythmic burden. Besides reservoir, conduct, and contractile functions, LA mediates cardiovascular homoeostasis through neurohormonal determinants that affect the electrophysiological properties of the atrial cardiomyocytes; LA remodeling in AF-related MR leads to loss of normal sympathoinhibition [31], which previous studies have documented in early phases after sinus rhythm restoration [32], but perhaps plays a pivotal role also in the long-term follow-up. However, some echocardiographic parameters, including LAVi, are not significantly different from baseline, which might be attributed to late referral [17,18].

Furthermore, at baseline, no differences in LA strain among patients with paroxysmal, persistent, and sinus rhythm were detected; this can be explained by the fact that LA function, per se, is not a primary element of steady state cardiac output [5]. However, there were differences between the subgroups at 6 and 12 months’ follow-up that become significant between sinus rhythm and permanent AF patients, since the first subgroup had a higher prevalence of reversible atrial changes in atrial function than the other subgroups.

Different MR etiology (primary, secondary, and mixed) did not affect LA strains at baseline nor during follow-up, probably because all the subgroups represent chronic clinical settings that cause similar structural alterations in terms of derangement and fibrosis deposition.

Finally, positive remodeling of LA and amelioration of LA function, especially in cases where structural alterations are not in the advanced phase, contributed to avoiding new-onset AF in patients with severe MR and in maintaining sinus rhythm, with the observed net result of reduced arrhythmic burden. One might speculate that there was a reduction in the sympathetic adrenergic overdrive that characterizes HF patients, including those candidates for TEER. Like chronic AF patients, arrhythmic burden of paroxysmal AF was found reduced in the current study, as well as a shorter duration of the episodes in persistent forms. In all cases, a more compensated state could have co-participated in cardiac rhythm stabilization, including heart rate reduction. On the other hand, it could be also considered that TEER heightens the cardiovascular response to pharmacologic agents in HF.

Among the main limitations of the study, it must be acknowledged that the retrospective nature and the relatively small sample size do not confer to this work a comprehensive vision of the independent impact of atrial mechanics on cardiovascular outcomes. Also, due to inherent limitations of the 2D-STE technique, it was not possible to conduct STE analysis in all patients considered at the beginning of the enrollment, since those cases with poor image quality, as well as those with a too-low frame rate or other artifacts, have been discarded. The duration of follow-up of one year might be underestimating more severe outcomes such as the risk of death and HF hospitalization. One last limitation might be due to the relative role of optimal medical therapy [33], which might have concurred with TEER in providing the above-elucidated clinical benefits.

## 5. Conclusions

TEER caused a favorable atrial remodeling in the months following MR reduction as it was detected by 2D-STE, which allowed highlighting an improvement in the reservoir phase (strain reservoir) and in the conduction strain in postintervention follow-up. The reservoir strain represents the most effective 2D-STE parameter that can be detected in early phases after TEER and LA function amelioration. Furthermore, LA-positive remodeling contributes to limiting the incidence of ventricular and atrial tachyarrhythmias, mostly AF. Accurate selection of cases before invasive procedures is mandatory for identifying those patients who could benefit more from multiple therapies such as TEER and electrical nonpharmacologic therapies for AF.

## Figures and Tables

**Table 1:** Baseline clinical characteristics.

Age (Mean ± SD ^1^, Years)	76 ± 6
Male gender, *n* (%)	64 (59)
hFpEF ^2^, *n* (%)	43 (39)
hFmrEF ^3^, *n* (%)	19 (18)
hFrEF ^4^, *n* (%)	47 (43)
Systemic hypertension, *n* (%)	94 (86)
Dyslipidemia, *n* (%)	72 (66)
Smoke, *n* (%)	10 (9)
COPD ^5^, *n* (%)	31 (28)
Diabetes, *n* (%)	37 (34)
Prior stroke, *n* (%)	9 (8)
Prior valvular surgery, *n* (%)	3 (2)
Prior myocardial infarction, *n* (%)	35 (32)
Prior revascularization, *n* (%)	49 (45)
CKD ^6^, *n* (%)	48 (44)
AF ^7^, *n* (%)	61 (56)
Paroxysmal AF, *n* (%)	18 (29)
Persistent AF, *n* (%)	9 (15)
Permanent AF, *n* (%)	34 (56)
Euroscore II (mean ± SD)	11.0 ± 11.3
STS ^8^ score (mean ± SD)	2.9 ± 1.9
Primary MR ^9^, *n* (%)	41 (38)
Secondary MR, *n* (%)	51 (47)
Mixed etiology, *n* (%)	17 (15)
MR severity at discharge	
1, *n* (%)	57 (52)
2, *n* (%)	47 (43)
3, *n* (%)	3 (3)
4, *n* (%)	2 (2)
CIEDs ^10^, *n* (%)	32 (29)
Pacemaker, *n* (%)	8 (25)
ICD ^11^, *n* (%)	17 (53)
CRT-D ^12^, *n* (%)	7 (22)

^1^ Standard deviation, ^2^ Heart failure with preserved ejection fraction; ^3^ Heart failure with mildly reduced ejection fraction; ^4^ Heart failure with reduced ejection fraction; ^5^ Chronic obstructive pulmonary disease; ^6^ Chronic kidney disease; ^7^ Atrial fibrillation; ^8^ Society of Thoracic Surgeons; ^9^ Mitral regurgitation; ^10^ Cardiac implantable electronic devices; ^11^ Implantable cardioverter–defibrillators; ^12^ Cardiac resynchronization therapy device with defibrillator.

**Table 2:** Baseline and 1-year following TEER ^1^ echocardiographic assessments.

Parameters	Baseline	Follow-Up	*p* Value
RIGHT VENTRICLE/RIGHT ATRIUM	Mean ± SD ^2^	Mean ± SD	
RV ^3^ GS ^4^ (%)	−13.0 ± 6.0	−15.5 ± 4.1	0.04
TAPSE ^5^ (mm)	19.4 ± 2.8	20.8 ± 5.0	0.08
PAPs ^6^ (mmHg)	46.9 ± 12.1	42.3 ± 10.7	0.02
RV diameter (mm)	39.1 ± 4.3	38.9 ± 3.7	0.62
LEFT VENTRICLE/LEFT ATRIUM			
LVEDD ^7^ (mm)	57.3 ± 7.6	51.7 ± 9.3	0.001
LV EF ^8^ (%)	44.1 ± 12.0	45.9 ± 11.0	0.04
LV GLS ^9^ (%)	−13.7 ± 7.9	−15.0 ± 7.9	0.04
LAVi ^10^ (ml/m^2^)	56.8 ± 17.3	56.7 ± 11.2	0.14
LAEF−4CH ^11^ (%)	28.4 ± 11.0	36.9 ± 18.7	0.03
R_s ^12^ (%)	10.7 ± 5.8	16.3 ± 6.8	<0.001
D_s ^13^ (%)	−6.7 ± 4.5	−9.6 ± 4.5	0.008
C_s ^14^ (%)	−5.2 ± 5.1	−7.8 ± 5.4	0.11

^1^ Transcatheter edge-to-edge repair; ^2^ Standard deviation; ^3^ Right ventricle; ^4^ Global strain; ^5^ Tricuspid annular plane systolic excursion; ^6^ Pulmonary artery systolic pressure; ^7^ Left ventricular end-diastolic diameter; ^8^ Ejection fraction; ^9^ Global longitudinal strain; ^10^ Left atrial volume index; ^11^ Left atrial emptying fraction—4 chambers; ^12^ LA reservoir strain; ^13^ LA conduct strain; ^14^ LA contractile strain.

**Table 3:** Atrial fibrillation progression after TEER ^1^.

	Baseline	12-Month FU ^2^	*p* Value
Burden (hours per day) in paroxysmal AF ^3^ (*n* = 18)	17.6 ± 6.0	12.3 ± 6.4	0.002
Duration (hours) in persistent AF (*n* = 9)	>168	16.2 ± 4.0	0.018
Ventricular rate (bpm ^4^) in permanent AF (*n* = 34)	78 ± 12	73 ± 13	0.006

^1^ Transcatheter edge-to-edge repair; ^2^ Follow-up; ^3^ Atrial fibrillation; ^4^ Beats per minute.

## Data Availability

Romano and Curcio had full access to all of the data in the study and take responsibility for the integrity of the data and the accuracy of the data analysis. The data underlying this article will be shared upon reasonable request to the corresponding author.
